# Fat intake and composition of fatty acids in serum phospholipids in a randomized, controlled, Mediterranean dietary intervention study on patients with rheumatoid arthritis

**DOI:** 10.1186/1743-7075-2-26

**Published:** 2005-10-10

**Authors:** Linda Hagfors, Ingela Nilsson, Lars Sköldstam, Gunnar Johansson

**Affiliations:** 1 Department of Food and Nutrition, Umeå University, SE-901 87 Umeå, Sweden; 2 Department of Clinical Chemistry, Kalmar County Hospital, SE-391 85 Kalmar, Sweden; 3 Department of Medicine, Visby Hospital, SE-621 84 Visby, Sweden

## Abstract

**Background:**

We have previously reported that rheumatoid arthritis patients, who adopted a modified Cretan Mediterranean diet, obtained a reduction in disease activity and an improvement in physical function and vitality. This shift in diet is likely to result in an altered intake of fatty acids. Therefore, the objective of the present study was to examine the dietary intake of fatty acids, as well as the fatty acid profile in serum phospholipids, during the dietary intervention study presented earlier.

**Results:**

From baseline to the end of the study, changes in the reported consumption of various food groups were observed in the Mediterranean diet group. The change in diet resulted in a number of differences between the Mediterranean diet group and the control diet group regarding the fatty acid intake. For instance, a lower ratio of n-6 to n-3 fatty acids was observed in the Mediterranean diet group, both assessed by diet history interviews (dietary intake) and measured in serum phospholipids. Moreover, the patients in the Mediterranean diet group that showed a moderate or better clinical improvement during the study (diet responders), had a higher reported intake of n-3 fatty acids and a lower ratio of n-6 to n-3 fatty acids compared to the patients with minor or no improvement. Also the fatty acid profile in serum phospholipids differed in part between the diet responders and the diet non-responders.

**Conclusion:**

The changes in the fatty acid profile, indicated both by dietary assessments and through fatty acids in s-phospholipids may, at least in part, explain the beneficial effects of the Cretan Mediterranean diet that we have presented earlier.

## Background

We have previously reported that a shift to a modified Cretan Mediterranean diet decreased the disease activity and improved the physical function and vitality of Swedish rheumatoid arthritis (RA) patients [1]. Among the characteristics of the experimental diet used in this study, was a relatively high consumption of fish, olive oil and canola (rapeseed) oil, as well as a low intake of other fats and of red meat. A change from a typical Swedish diet to a Cretan Mediterranean diet is likely to result in a changed intake of fatty acids, for example an increased intake of n-3 fatty acids. The impact of dietary fatty acids on rheumatoid inflammation has been investigated in a number of in-vitro and animal studies, as well as in randomized, placebo-controlled trials [2]. The fatty acids most thoroughly studied in relation to RA, are the n-3 fatty acids eicosapentaenoic acid (EPA; 20:5n-3) and docosahexaenoic acid (DHA; 22:6n-3). At least thirteen randomized controlled trials, ranging from 12 to 52 weeks, have shown that supplementation with fish oil (which contains EPA and DHA) resulted in beneficial effects on RA symptoms [3,4]. The most commonly observed benefit is a decreased number of tender joints [5], but improvements in morning stiffness, number of swollen joints, pain index, physicians global assessment and grip strength have also been reported [4]. In addition, a meta-analysis, as well as a mega-analysis (in which the original data sets were analysed), confirmed the efficacy of fish oils in reducing RA symptoms [6]. However, only the reduction in the tender joint count and in morning stiffness was shown to be statistically significant using these approaches.

In supplementation studies, olive oil has sometimes been used as a placebo oil. In some of these studies improvements were reported also in the group of RA patients treated with olive oil [7-9]. The exact mechanism for this effect has been uncertain. However, in a recent study, a compound in extra-virgin olive oil, called oleocanthal, was shown to inhibit the activity of cyclooxygenase enzymes [10].

The relationship between n-3 fatty acids/fish intake, olive oil and RA, has also been observed in case control studies. In these studies a high consumption of fish [11] and olive oil [12,13] was associated with decreased risk of developing RA.

In view of the results from earlier studies, the beneficial effects observed in the randomized controlled trial in which the Cretan Mediterranean diet was tested [1], may be caused by an increse in n-3 fatty acids in relation to n-6 fatty acids and/or an increased intake of olive oil. The aim of the present study was to examine the consumption of food items with relevance to the fat intake, the dietary intake of fatty acids, as well as the fatty acid profile in serum phospholipids, in the subjects participating in this study.

## Results

### Reported dietary intake

To estimate the reported consumption of foods with relevance to the fatty acid intake, a self-administrated questionnaire was used. At baseline the Cretan Mediterranean diet (MD) group (n = 26) and the control diet (CD) group (n = 25) reported a similar intake frequency of all the food groups investigated (Table 1). By the end of the study the MD group had decreased their intake of several food groups such as meat, processed meat (including cured meat, sausage, pâté or the like), sweets and high fat dairy products, while the intake of fish, shellfish, poultry and cheese with less than 17% fat, had increased compared to baseline. The CD group also decreased their intake of meat, from baseline to the end of the study, but apart from that, no differences were seen in that group regarding the intake frequencies of foods with relevance to the fatty acid intake.

**Table 1:** Comparison of reported food consumption frequencies (servings per month) between the Mediterranean Diet (MD) group and the Control Diet (CD) group.

	**MD group (n = 26)**			**CD group (n = 25)**			P-value*
			
	**Time (weeks after dietary shift)**			**Time (weeks after start of study)**			
	**0**	**3**	**12**	**0**	**3**	**12**	
Fish	6 (6-6)	16 (6–16)	16 (16-16)^§^	6 (2–6)	6 (6–16)	6 (2–6)	<0.001
Shellfish	2 (0–2)	2 (0–6)	4 (2–6)^‡^	2 (0–2)	2 (0–2)	2 (0–2)	<0.001
Meat, minced meat or offal from pig, cattle or sheep	16 (6–16)	0 (0-0)	0 (0-0)^§^	16 (6–16)	6 (6–16)	6 (6–16)^†^	<0.001
Poultry	2 (2–6)	16 (6–16)	11 (6–16)^§^	2 (2–6)	2 (2–6)	2 (2–6)	<0.001
Processed meat^¶^(not on bread)	6 (2–6)	0 (0-0)	0 (0-0)^§^	6 (2–6)	6 (2–6)	2 (2–6)	<0.001
Processed meat^¶^on bread	11 (6–26)	0 (0-0)	0 (0-0)^§^	16 (2–16)	6 (4–16)	6 (2–16)	<0.001
Cheese with more than 17% fat	16 (14–26)	0 (0-0)	0 (0-0)^§^	26 (11–70)	16 (4–26)	16 (4–26)	0.002
Cheese with less than 17% fat	6 (0–9)	16 (0–26)	16 (6–26)^‡^	0 (0–4)	0 (0–6)	0 (0–11)	0.005
Dairy products with 27% fat or more	2 (0–6)	0 (0-0)	0 (0-0)^‡^	2 (0–6)	2 (0–6)	6 (2–6)	<0.001
Dairy products with 10–15% fat	2 (0–6)	0 (0-0)	0 (0–2)^‡^	2 (0–6)	0 (0–6)	0 (0–6)	0.013
Ice cream	2 (0–6)	0 (0–0.5)	0 (0–2)^‡^	2 (0–4)	2 (0–2)	2 (0–2)	0.015
Sweets (including chocolate)	6 (2–16)	0 (0–2)	0 (0–2)^§^	6 (1–6)	6 (0–6)	6 (2–6)	<0.001
Buns, cookies or cakes	6 (5–16)	0 (0–2)	0 (0–2)^§^	6 (2–16)	6 (2–16)	6 (2–16)	<0.001
Nuts	0 (0–2)	0 (0–2)	0 (0–3)	0 (0–2)	0 (0–2)	0 (0–2)	0.704
Seeds	0 (0-0)	0 (0–2)	0 (0–2)	0 (0-0)	0 (0-0)	0 (0-0)	0.016

Data are presented as medians (25th–75th percentiles). *The p-values refer to differences between the MD- and the CD group concerning the change from baseline to week 12. Differences between the groups were analyzed with the Mann-Whitney U test. Statistically significant change from baseline to week 12: †p < 0.05; ‡p < 0.01; §p < 0.001. Within-group differences in week 12 compared to baseline were evaluated by the Wilcoxon signed ranks test. ¶Including cured meat, sausage, pâté or the like.

Regarding the reported food choices, significantly more MD subjects than CD subjects changed their choice of milk and fermented milk to milk and fermented milk with a lower fat content, or excluded these foods totally (p = 0.003 and p = 0.038 respectively). In addition, more MD subjects than CD subjects used olive oil and canola oil, as well as the liquid margarine and half-fat margarine supplied during the study period (p < 0.001 for all comparisons).

The nutrient intake during the second half of the study was estimated by means of diet history interviews (conducted with 17 individuals in each group). Two persons in the CD group and one in the MD group were identified as under-reporters, based on the individual Goldberg cut-off [14] for the food intake level (FIL). In addition, one CD subject was identified as an over-reporter. Since the results at the group level were unchanged, whether we excluded under- and over-reporters or not, the results of all the diet history interviews are presented (Table 2). One exception was however, the percentage of energy (E%) derived from polyunsaturated fatty acids (PUFAs), which was not significantly different between the groups when under- and over-reporters were excluded.

**Table 2:** Comparison of average daily intake (excluding supplements) of energy, fat and specific fatty acids between the Mediterranean Diet (MD) group and the Control Diet (CD) group. The dietary intake is based on the diet history interviews performed between study weeks seven and twelve.

	**MD group **(n = 17)	**CD group **(n = 17)	P-value*
Energy (MJ)	8.8 ± 1.6	9.8 ± 3.2	p = 0.242
Fat (g)	60.4 ± 21.9	89.3 ± 33.1	p = 0.005
Total saturated fatty acids (g)	18.3 ± 8.2	40.5 ± 18.3	p < 0.001
Total monounsaturated fatty acids (g)	25.3 ± 10.5	31.9 ± 11.1	p = 0.088
Total polyunsaturated fatty acids (g)	11.8 ± 3.9	10.6 ± 3.5	p = 0.381
Total n-6 fatty acids (g)^†^	7.9 ± 2.5	8.2 ± 2.7	p = 0.743
Total n-3 fatty acids (g)^‡^	3.1 ± 1.3	2.0 ± 0.9	p = 0.008
Ratio n-6:n-3	2.7 ± 0.6	4.4 ± 0.9	p < 0.001
Fat (E%^§^)	25.0 ± 5.3	33.7 ± 5.6	p < 0.001
Total saturated fatty acids (E%)	7.5 ± 2.4	15.0 ± 3.7	p < 0.001
Total monounsaturated fatty acids (E%)	10.5 ± 2.8	12.2 ± 2.2	p = 0.067
Total polyunsaturated fatty acids (E%)	5.0 ± 1.1	4.1 ± 1.1	p = 0.028^¶^
*Fatty acids (g):*			
4:0–10:0	0.77(0.32–1.59)	3.51(1.72–4.79)	p < 0.001
12:0	0.44(0.28–0.93)	1.67(0.93–2.56)	p < 0.001
14:0	1.81(1.07–2.90)	4.68(2.79–6.19)	p < 0.001
16:0	9.46(7.29–13.29)	18.54(14.98–26.00)	p < 0.001
18:0	2.80(1.99–4.34)	6.85(5.94–9.97)	p < 0.001
20:0	0.14(0.10–0.19)	0.19(0.16–0.38)	p = 0.016
16:1n-7	0.95(0.70–1.22)	1.25(1.05–1.84)	p = 0.014
18:1n-9	20.50(14.98–28.05)	27.55(21.52–35.67)	p = 0.049
18:2n-6	7.42(5.80–9.46)	7.90(5.78–9.77)	p = 0.892
18:3n-3	1.79(1.23–2.36)	1.42(1.09–2.09)	p = 0.454
20:4n-6	0.08(0.05–0.10)	0.08(0.04–0.13)	p = 0.708
20:5n-3	0.35(0.23–0.59)	0.11(0.06–0.18)	p < 0.001
22:5, n-3 and n-6	0.07(0.05–0.11)	0.02(0.01–0.05)	p = 0.001
22:6n-3	0.73(0.44–1.03)	0.21(0.12–0.30)	p < 0.001

Data are presented as mean ± SD for normally distributed variables and as medians (25th–75th percentiles) for variables with skew distributions. *The P-values refer to the difference between diet- and control group. Differences between groups were analyzed by the Students t-test for independent samples for normally distributed variables and by the Mann-Whitney U test for variables with skew distributions; †sum of 18:2n-6 and 20:4n-6; ‡sum of 18:3n-3, 20:5n-3 and 22:6n-3; §E% = percent of total energy. ¶Difference between groups regarding E% polyunsaturated fatty acids was not significant (p = 0.101) when under- and over-reporters were excluded.

According to the results from the diet history interviews, the MD group had a lower intake of fat and saturated fatty acids (SFAs), compared to the CD group (Table 2). Although there was no significant difference between the groups regarding the total intake of monounsaturated fatty acids (MUFAs), the intake of both oleic acid (OA; 18:1n-9) and palmitoleic acid (16:1n-7) was higher in the CD group. Regarding PUFAs, the reported intake of n-6 fatty acids did not differ significantly between the groups, while the intake of the long-chain n-3 fatty acids was higher in the MD group. In this group the total intake of the n-3 fatty acids (18:3n-3, 20:5n-3 and 22:6n-3) was 3.1 g/day (of which EPA+DHA constituted 1.2 g/day), versus 2.0 g/day (EPA+DHA = 0.4 g/day) in the CD group. This difference in turn resulted in a significantly lower ratio of ingested n-6 to n-3 fatty acids in the MD group. Conversely, there was no significant difference between the groups regarding the absolute intake (g/day), or in E%, of α-linolenic acid (α-LNA; 18:3n-3) and linoleic acid (LA; 18:2n-6). In the MD group, the E% α-LNA was 0.80 and the E% LA was 3.3, versus 0.62 E% α-LNA and 3.1 E% LA acid in the CD group. However, as a consequence of the difference between the groups regarding the total fat intake, both α-LNA, LA and OA contributed to a higher proportion of the fat intake in the MD group (mean intake of α-LNA, LA and OA expressed as g/100 g total fat was 3.2, 13.3 and 37.4 in the MD group, and 1.9, 9.4 and 32.7 in the CD group, p < 0.001, p < 0.001 and p = 0.012, respectively).

### Fatty acid composition in serum phospholipids

At baseline there were no significant differences between the groups regarding the composition of fatty acids in serum phospholipids (Table 3). From baseline to the end of the study the percentage of the saturated fatty acid arachidic acid (20:0) increased slightly in the MD group. Increased relative amounts were also observed in the MD group regarding the n-3 fatty acids EPA and DHA, and for the total percentage of n-3 fatty acids. As regards n-6 fatty acids, there was a decrease in the relative amount of dihomo-γ-linolenic acid (20:3n-6) and in the total percentage of n-6 fatty acids in the MD group. The differences in the proportions of n-6 and n-3 fatty acids in this group, resulted in a decrease in the n-6:n-3 ratio. In the CD group there were no significant changes in any of the fatty acids analyzed. When the correlation between the reported intakes of individual fatty acids and their relative amounts in s-phospholipids was calculated, both the relative intake of EPA (g/100 g total fat) and the absolute intake of EPA (g/day) were significantly correlated to the percentage of EPA in s-phospholipids (Table 4). Also the relative and absolute intakes of DHA were related to the percentage of EPA in s-phospholipids. Regarding α-LNA there was a significant correlation between the absolute intake of α-LNA (g/day) and the level of α-LNA in s-phospholipids.

**Table 3:** Comparison of the fatty acid composition (per cent of total fatty acids) in serum phospholipids between the Mediterranean Diet (MD) group and the Control Diet (CD) group.

	**MD group (n = 26)**			**CD group (n = 25)**			P-value*
			
	**Time (weeks after dietary shift)**			**Time (weeks after start of study)**			
	**0**	**3**	**12**	**0**	**3**	**12**	
16:0	28.3 (23.6–31.0)	29.0 (24.6–32.2)	27.8 (25.3–31.3)	28.9 (27.2–31.7)	29.4 (27.1–32.3)	29.8 (26.6–32.1)	0.797
18:0	14.5 (13.1–15.2)	13.7 (12.2–15.3)	13.7 (12.4–15.7)	15.0 (13.2–17.1)	14.6 (13.3–16.5)	15.0 (13.3–17.0)	0.584
20:0	0.0 (0.0–0.1)	0.0 (0.0–0.2)	0.0 (0.0–0.3)^†^	0.0 (0.0–0.2)	0.0 (0.0–0.2)	0.0 (0.0–0.2)	0.100
24:0	1.4 (1.0–2.2)	1.4 (1.0–1.9)	1.6 (1.1–2.0)	1.4 (1.0–1.6)	1.1 (0.8–1.7)	1.3 (0.8–1.8)	0.959
**Total saturated fatty acids**	44.0 (39.1–46.8)	44.7 (37.8–48.3)	43.9 (39.6–49.4)	45.6 (41.4–49.9)	45.6 (41.1–49.1)	46.0 (42.7–49.9)	0.705
16:1n-7	0.8 (0.6–0.9)	0.7 (0.6–0.8)	0.8 (0.6–0.9)	0.7 (0.6–1.1)	0.8 (0.6–0.9)	0.8 (0.6–1.0)	0.339
18:1n-7	1.7 (1.3–1.8)	2.0 (1.4–2.2)	1.8 (1.6–2.0)	1.7 (1.3–1.9)	1.7 (1.5–2.1)	1.8 (1.5–2.0)	0.557
18:1n-9	9.9 (8.2–10.9)	9.7 (8.3–11.2)	9.4 (7.3–10.6)	10.2 (8.3–11.1)	10.6 (8.9–11.5)	10.9 (8.3–11.5)	0.087
22:1n-11	0.6 (0.2–1.6)	0.6 (0.2–1.5)	0.8 (0.8–1.8)	0.6 (0.0–1.3)	0.3 (0.0–1.0)	0.6 (0.0–1.1)	0.356
**Total monounsaturated fatty acids**	13.1 (11.4–13.8)	12.8 (11.2–14.6)	12.6 (11.3–13.8)	13.3 (11.8–14.4)	13.6 (11.6–14.7)	13.9 (11.4–15.0)	0.207
18:2n-6	16.7 (11.5–19.9)	16.5 (10.7–18.6)	14.4 (11.0–16.9)	16.0 (12.5–19.2)	16.7 (12.8–18.1)	17.0 (13.5–19.5)	0.044
20:2n-6	0.4 (0.2–0.7)	0.3 (0.2–0.5)	0.4 (0.2–0.6)	0.3 (0.2–0.5)	0.3 (0.2–0.5)	0.4 (0.3–0.6)	0.458
20:3n-6	2.6 (1.8–3.2)	2.5 (1.7–3.0)	2.2 (1.6–2.6)^‡^	2.1 (1.5–3.1)	2.2 (1.7–2.8)	2.4 (1.8–3.1)	0.003
20:4n-6	6.9 (5.0–7.7)	6.8 (4.8–8.2)	6.6 (4.1–8.0)	5.9 (4.1–9.8)	6.6 (4.2–8.5)	6.5 (4.8–8.7)	0.144
**Total n-6 fatty acids**	25.2 (18.9–30.7)	26.8 (17.5–30.0)	23.8 (17.6–28.3)^†^	24.4 (18.4–32.2)	25.1 (19.3–29.9)	25.2 (21.5–30.5)	0.008
18:3n-3	0.3 (0.0–0.4)	0.2 (0.0–0.4)	0.4 (0.0–0.4)	0.3 (0.2–0.4)	0.3 (0.1–0.5)	0.3 (0.0–0.5)	0.395
20:5n-3	1.4 (1.1–1.8)	1.8 (1.1–2.8)	2.1 (1.4–3.3)^§^	1.1 (1.0–1.5)	1.1 (1.0–1.7)	1.1 (0.9–1.4)	<0.001
22:5n-3	0.7 (0.2–1.7)	0.6 (0.2–1.4)	0.8 (0.2–1.7)	0.5 (0.2–1.3)	0.6 (0.2–1.0)	0.6 (0.3–1.0)	0.395
22:6n-3	3.9 (2.2–4.8)	4.2 (3.1–6.6)	5.0 (2.5–7.2)^§^	3.0 (1.5–4.3)	3.5 (1.7–4.2)	3.0 (2.2–4.5)	<0.001
**Total n-3 fatty acids**	6.1 (5.2–7.4)	7.3 (5.3–10.0)	8.6 (6.0–11.7)^§^	5.2 (4.3–7.0)	4.9 (3.7–6.4)	5.2 (3.8–7.0)	0.001
**n-6:n-3 ratio**	4.2 (3.0–5.6)	3.1 (2.7–3.7)	2.5 (2.1–3.5)^‡^	4.6 (3.6–5.6)	4.4 (3.4–5.6)	4.9 (4.1–5.6)	0.002
**Total polyunsaturated fatty acids**	32.7 (25.9–38.4)	33.5 (24.4–40.1)	32.4 (22.7–39.0)	29.3 (22.3–37.5)	31.0 (24.0–36.6)	30.9 (25.7–37.4)	0.072

Data are presented as medians (25th–75th percentiles). *The p-values refer to differences between the MD- and the CD group concerning the change from baseline to week 12. Differences between the groups were analyzed with the Mann-Whitney U test. Statistically significant change from baseline to week 12: †p < 0.05, ‡p < 0.01, §p < 0.001. Within-group differences in week 12 compared to baseline were evaluated by the Wilcoxon signed ranks test.

**Table 4:** Spearman's correlation coefficients between the reported intake of fatty acids or foods and fatty acids in serum phospholipids.

	Fatty acids in s-phospholipids			
	
Dietary fatty acids/foods	EPA	DHA	α-LNA	total n-3
EPA g/day	0.58*	0.24	0.04	0.43^†^
EPA g/100 g total fat	0.58*	0.32	0.04	0.49*
DHA g/day	0.60*	0.19	-0.05	0.40^†^
DHA g/100 g total fat	0.57*	0.26	-0.09	0.45*
α-LNA g/day	0.13	-0.09	0.38^†^	0.03
α-LNA g/100 g total fat	0.24	0.11	0.29	0.20
Change in fish intake^‡^	0.48*	0.46*	0.14	0.51*
Change in shellfish intake^‡^	0.33^†^	0.48*	0.03	0.34^†^

*p < 0.01; †p < 0.05; ‡change from baseline to week 12 in relation to change in percentage of fatty acids in s-phospholipids.

Furthermore, changes in the reported consumption frequencies of fish and shellfish were positively related to changes in EPA, DHA and the total percentage of n-3 fatty acids in s-phospholipids (Table 4).

### Differences between diet responders and diet non-responders

When the MD group was divided into diet responders (n = 15) and diet non-responders (n = 11), based on their clinical improvement during the study, the diet responders had a higher reported relative intake (fatty acids expressed in relation to the total fat intake) of EPA, docosapentaenoic acid (22:5, which in the food composition database used includes both n-3 and n-6 isomers) and DHA compared to the diet non-responders (p = 0.032, p = 0.040 and p = 0.011 respectively). This resulted in a lower intake ratio of n-6 to n-3 fatty acids in the group of diet-responders. The median n-6 to n-3 ratio was 2.2 in the diet responders and 3.0 in the diet non-responders (p = 0.032). Also the intake of the MUFA palmitoleic acid (16:1n-7), in relation to the total fat intake, was higher in the diet responders (p = 0.015), while the E% of total MUFAs was higher in the diet non-responders (p = 0.048). However, when the intake of total MUFAs was expressed in relation to the total fat intake there was no significant difference between the groups (p = 0.380).

Regarding the fatty acid composition in serum phospholipids there were some differences between the diet responders and the diet non-responders at baseline. The diet responders had a higher relative amount of lignoceric acid (24:0) and lower relative amounts of arachidic acid (20:0), vaccenic acid (18:1n-7) and α-LNA (p = 0.042, p = 0.022, p = 0.007 and p = 0.024 respectively). The change in fatty acid composition from baseline to the end of the study also differed between the groups. The relative amounts of the SFAs palmitic acid (16:0) and stearic acid (18:0), as well as the total SFAs, increased in the diet non-responders, while these fatty acids decreased in the diet responders (p = 0.040, p = 0.033 and p = 0.007 respectively for differences between the groups). On the other hand, the n-3 fatty acid docosapentaenoic acid (22:5n-3) increased in the diet responders while a slight decrease was observed in the diet non-responders (p = 0.048). Regarding the other n-3 fatty acids in serum phospholipids, no significant differences were found between the groups.

No significant differences between the groups were observed concerning the reported consumption of food groups. However, from baseline to the end of the study there was a tendency towards a greater increase in the fish consumption (p = 0.059), as well as a greater decrease in the consumption of dairy products with 10–15% fat (p = 0.076), in the group of diet responders compared to the non-responders.

## Discussion

### Dietary intake of fat and specific fatty acids

In this study we used a questionnaire to estimate the reported consumption of foods with relevance to the fatty acid intake, and diet history interviews to assess the intake of fat and specific fatty acids. In the MD group, the changes in the reported consumption of food items were in line with the advice given to the patients, i.e. to replace meat, and processed meat with fish and poultry, to use olive oil, canola oil or margarine based on canola oil, and to choose low fat dairy products. This was also reflected in the reported intake of fat assessed by the diet history interviews. For instance, choosing low fat dairy products instead of higher fat alternatives is likely to result in a decreased intake of both total fat and of SFAs. Thus, both assessments of dietary intake indicated that the MD group changed their dietary intake in the direction aimed at.

The traditional Cretan Mediterranean diet is well known for its high content of MUFAs from olive oil. According to a study by Cleland at al, the incorporation of EPA into neutrophil membranes after fish-oil supplementation was higher in healthy subjects eating a diet high in OA and low in LA, compared to subjects on a diet high in LA [15]. Thus, when the dietary intake of OA is high in relation to LA, this may be of advantage to the n-3 fatty acids in the competition between n-6 and n-3 fatty acids. In this study we also used canola oil which, similar to olive oil, is rich in the MUFA OA. Nevertheless, there was a tendency towards a lower intake of MUFAs in the MD group (p = 0.088). This was probably a result of the considerably lower intake of total fat in this group, since the relative amount of OA was still significantly higher than in the CD group.

Among the most important characteristics of the experimental diet was the amount of n-3 fatty acids and the relation between n-6 and n-3 fatty acids. The original Cretan Mediterranean diet was rich in n-3 fatty acids from both animal sources and plant sources [16]. In the present study, the MD group was instructed to use canola oil and margarine based on canola oil, which are good sources of α-LNA. This, together with an increased intake of fish and shellfish, resulted a significantly lower ratio of n-6:n-3 fatty acids in the MD group (2.7 versus 4.4). The n-6:n-3 ratio has been reported to be 1–2 in the traditional diet of Greece, while the ratio in many Western countries is around 15–17 [17]. However, in the Swedish nationwide dietary survey "Riksmaten 1997–98" [18], where the patterns of food and nutrient intake in Sweden were studied on a nationwide basis, the ratio n-6:n-3 was reported to be around 5. This is not far from the ratio in our CD group, while the MD group obtained a ratio closer to that of the traditional Greek diet. However, it is important to remember, that when data from different countries and different studies are compared, different fatty acids may have been used to calculate these ratios.

A modified Cretan Mediterranean diet, similar to our experimental diet, has been tested by de Lorgeril et al [19,20], in a study on secondary prevention of coronary heart disease. In that study the aim was to reduce the intake of LA to 4 E% and the intake of α-LNA was to exceed 0.6 E%. After eight weeks the experimental group in their study had an LA intake of 3.6 E% and an α-LNA intake of 0.76 E%. In the present study, the E% of LA was even lower and the E% of α-LNA was almost the same in the MD group as in the study by de Lorgeril et al. However, the intake of LA and α-LNA in our CD group was not very different from the MD group. This may be explained by the relatively high use of canola oil and margarine based on canola oil in Sweden. Although we encouraged our MD group to increase the intake of these α-LNA sources, these foods are also important sources of n-3 fatty acids in the Swedish population in general [18]. Thus, regarding essential fatty acids, even the intake of the CD group was close to that of the experimental group in the study by de Lorgeril et al [20].

In the present study, the reported total intake of n-3 fatty acids in the MD group was 3.1 g/day and of these 1.2 g were EPA + DHA. Most studies of dietary supplementation with fish oil have used between 1 and 7.1 g EPA + DHA per day [2]. On the basis of these studies, a daily intake of 3–6 g long-chain n-3 fatty acids (usually EPA + DHA) has been recommended in order to improve RA symptoms [5,21]. Hence, although the MD group reported an increased consumption of fish, the intake of long chain n-3 PUFAs only reached the lower range of the amounts used in studies of fish oil supplementation. However, in these studies n-3 fatty acids are usually added to the diet, regardless of the intake of other fatty acids. In a few supplementation studies the amount of competitor n-6 fatty acids has been considered as well [3,22,23]. Volker et al [3] investigated the effect of fish oil supplementation (40 mg/kg body weight/day) in RA subjects with a background diet of <10 g n-6 fatty acids. After 15 weeks, the experimental group achieved substantial incorporation of EPA in plasma and monocyte phospholipids, and significant improvements in six of nine outcome measures. Furthermore, in a study by Adam et al [22] the effect of fish oil supplementation (30 mg/kg body weight/day) in two groups on different diets was studied. One group were on a diet low in arachidonic acid (AA; 20:4n-6; less than 90 mg/day) and the other group ate a normal western diet. Patients from both groups were randomized to receive either fish oil capsules or placebo capsules for three months, in a double-blind, crossover manner. The results of the study showed that patients on the AA-low diet improved more than the other group regarding tender and swollen joints. As the authors conclude, this indicates that the ratio of AA:EPA is decisive for the clinical effectiveness of fish oil supplementation. In the present study both groups reported a daily intake of less than 10 g of n-6 fatty acids, and less than 90 mg AA. Based on the studies mentioned above, the low background intake of n-6 fatty acids may have amplified the effect of the Mediterranean diet.

### Fatty acid composition in serum phospholipids

The dietary intake of fat is often difficult to assess by means of dietary assessment methods. Therefore, there is a need for objective markers of fatty acid intake to be used as a complement to dietary assessment methods [24,25]. In the present study, we measured fatty acids in serum phospholipids, which are influenced by the dietary intake of the past few days [26]. The changes we found regarding the fatty acid profile were in agreement with the reported nutrient intake, i.e. an increase in the relative amount of n-3 fatty acids. We also observed a decrease in n-6 fatty acids in serum phospholipids. However, since an increased relative amount of one fatty acid cause a decrease in the percentage of another, this is not necessarily a consequence of a decreased intake of n-6 fatty acids. Somewhat surprisingly, the saturated fatty acid arachidic acid (20:0) increased marginally, but significantly, in the MD group. One possible reason for this could be an increased consumption of canola oil. Although the amount of arachidic acid is low in canola oil, this oil contains more arachidic acid than other oil, margarine or butter.

The use of fatty acid patterns in serum phospholipids as biomarkers of the dietary intake is, limited by the fact that not only diet affects the levels of fatty acids in biological specimens [24]. For instance, most fatty acids can be synthesized by humans and, within the body, fatty acids can also be converted to other fatty acids by means of desaturation and elongation. Furthermore, the pattern of plasma fatty acids in phospholipids has been reported to be altered in RA patients compared to healthy subjects [27]. In this study, the strongest correlation between the intake of fatty acids and the corresponding biomarkers, was observed regarding EPA (r_s _= 0.58 for both absolute and relative intakes). Similar, as well as both stronger and weaker correlations have been reported by others, possibly due to variations in the quality of the dietary data [28-30]. In other studies the percentage of DHA in phospholipids has been correlated to the intake of this fatty acid [28,29], while in the present study, the intake of DHA was only correlated to the percentage of EPA in phospholipids. The reason for this is probably that the dietary sources of EPA and DHA are the same. Also a significant correlation between α-LNA in serum phospholipds and the absolute intake of this fatty acid (r_s _= 0.38) was found in the present study. This result is partly in line with a study by Sasaki et al [31], while others have reported only weak correlation regarding α-LNA [29,30].

Sometimes, the relative fatty acid levels reflect the intake of specific foods, especially when the food item is the major source of the fatty acid in question. In this study changes in the reported consumption of fish and shellfish were related to changes in the percentage of both EPA, DHA and total n-3 fatty acids, with Spearman's correlation ranging from 0.46 to 0.51 for fish and from 0.33 to 0.48 for shellfish. Regarding fish intake, this is in agreement with other studies [28,32,33]. The correlation between shellfish intake and long-chain n-3 fatty acids may also be related to the consumption of fish. Although the reported intake of shellfish increased significantly in the MD group, the level of shellfish consumption was not very high in this study. Thus, compared to the intake of fish, shellfish was probably not a major source of n-3 fatty acids. Furthermore, there was a significant correlation between the change in fish intake and the change in shellfish intake (data not shown), indicating that the individuals reporting an increased intake of shellfish are likely to be the same individuals as those with an increased fish intake.

### Can the altered intake of fatty acids explain the beneficial effects on RA disease activity?

To analyse the possible connection between the altered intake of fatty acids in the MD group and the improvement regarding disease activity, we divided the MD group into diet responders and diet non-responders, based on the individual change in disease activity during the study. It is important to remember that only 15 (diet responders) and 11 (diet non-responders) patients were compared in this analysis. Hence, the power to detect differences between diet responders and diet non-responders was fairly low. Still, when these two groups were compared differences in both the reported intake of fatty acids and the fatty acid composition in serum phospholipids were found. Overall, the results point towards a more favourable fatty acid intake in the group of diet responders, at least regarding PUFAs, which in turn suggests a better compliance to the experimental diet in this group. Thus, the differences found between diet responders and diet non-responders indicate that the fatty acid intake contributed to the positive effect of this diet on RA disease activity.

## Conclusion

In conclusion, the changes in the reported consumption of food items in the MD group were in line with the advice given during the study. As a result, the total fat intake was lower in the MD group compared to the CD group, and in the MD group a lower percentage of the energy intake was derived from SFAs. The MD group also had a lower intake ratio of n-6:n-3 fatty acids. A corresponding change in the relation between n-6 and n-3 fatty acids was observed in s-phospholipids. Furthermore, the MD patients who were characterised as diet responders, had a higher reported intake of long chain n-3 fatty acids and a lower ratio of n-6 to n-3 fatty acids compared to the diet non-responders. Also the fatty acid profile in serum phospholipids differed in part between the diet responders and the diet non-responders. These findings point towards a better compliance to the experimental diet in the group of diet responders.

Altogether, the changes in the fatty acid profile indicated by three methods, a questionnaire, diet history interviews and fatty acids in s-phospholipids may, at least in part, explain the beneficial effects on the clinical measurements demonstrated earlier [1]. Thus, the results of this study support previous studies indicating the importance of the fatty acid intake in patients with RA.

## Methods

### Patients, study design and the experimental diet

The study was a randomised, parallel, dietary intervention study over three months. In total, 56 patients with RA were included in the study. Of these, 26 MD subjects (21 women and 5 men, mean age 58 years) and 25 CD subjects (20 women and 5 men, mean age 59 years) completed the study. The patients, study design and the dietary intervention have been described in detail elsewhere [1,34]. In brief, the patients were randomized to either a modified Cretan Mediterranean diet group or a control diet group, by means of block randomization stratified for sex. At baseline the two groups were equal except for the disease duration and the body mass index (BMI). The MD group had a significantly higher BMI and a longer disease duration compared to the CD group (p = 0.024 and 0.047, respectively).

The experimental diet used in the present study was based on the Cretan Mediterranean diet previously tested by de Lorgeril et al, in a secondary prevention study of coronary heart disease [19]. However, some modifications of the diet were done in order to suit Swedish food habits. We instructed our MD group to eat a large amount of vegetables, fruit, pulses, cereals, fish (particularly fish with a high content of ω-3 fatty acids) and nuts and seeds with a high content of α-LNA. The intake of meat (such as pork, beef, lamb or mutton) and processed meat (including cured meat, sausage, pâté or the like) were to be replaced by poultry, fish or vegetarian dishes. Both olive oil and canola oil were used in salad dressings and for food preparation. The MD group was also informed to use two types of margarine based on canola oil: a liquid margarine (80% fat) for food preparation and half-fat margarine (40% fat) to use on bread. In addition, the MD group was advised to replace high fat dairy products with low fat products. In the present study, no recommendations were given regarding alcohol consumption. To compensate for the antioxidants in wine, we advised the MD group to drink green or black tea.

To promote good compliance with the Mediterranean diet some food items were supplied free to the MD group, namely: frozen vegetables, tea, olive oil, canola oil and the liquid and half-fat margarine based on canola oil. Olive oil and canola oil, were supplied by Karlshamns AB, vegetables by Nestlé Sweden AB and margarine and tea by Van den Bergh Foods AB.

The CD subjects were instructed to adhere to their ordinary diet. If the subjects of the study used any dietary supplementation (e.g. fish oils, vitamins, minerals, etc.) prior to the study this was recorded. All such supplementation had to be kept unchanged during the study.

### Dietary assessments

To assess the dietary intake both a self-administered questionnaire and diet history interviews were used. The questionnaire was completed by the patients of both groups at baseline, in week three and twelve. This questionnaire included both open and closed questions, mainly concerning food choices, and it was designed to investigate compliance with the Mediterranean diet. Regarding questions on frequencies of food intake, the subjects should state their average intake of various food items by marking one of six alternatives ranging from "rarely or never" to "two or more times per day". To enable comparisons of the consumption between the two groups, as well as consumption at different points in time, the food frequencies were converted to average consumption per month. For example, if a consumption of 3–5 times per week was marked this would be converted to 16 times per month.

Diet history interviews, covering the dietary intake during the past month, were carried out to obtain more detailed data on the energy and nutrient intake during the intervention period. The interviews were performed between study weeks seven and twelve and were conducted with 34 patients from both the MD group and the CD group (15 women and 2 men from each group). The only selection criterion for taking part in the diet history interviews was that the subjects were included in the study on February 15^th ^1999, or later. Fore more details regarding the questionnaire and the diet history interviews see reference 34.

The nutritional analysis package MATs 4_03e was used to calculate the estimated intake of energy and nutrients based on the diet history interviews. This program is based on the Swedish National Food Administration's food composition database, PC-kost (version 2_97). If composite food items and supplements not listed in the database were reported, the nutrient content was entered manually. The energy and nutrient intake was calculated both including and excluding dietary supplements. Still, when dietary supplements were included, the total intake of n-3 fatty acids increased marginally in the MD group and not at all in the CD group. Therefore, only the dietary intake without supplements will be presented. However, when the correlation between the reported dietary intake of fatty acids and their relative content in s-phospholipids were analyzed, the results from the diet history interviews *including *supplements were used.

### Validation of the diet history interviews

A validation of the diet history interviews by means of the doubly labelled water method (performed for nine subjects) and biological markers of the intake of protein, sodium and potassium has been presented elsewhere [35]. To identify under- and over-reporters we used the individual Goldberg cut-offs for the FILs [14]. These cut-offs are based on the physical activity level for each subject assessed by a three-day activity registration, which has been described earlier [35]. The FIL is the individuals reported energy intake divided by the estimated basal metabolic rate. The basal metabolic rate was estimated based on body weight, age group and sex, according to standard equations [36].

### Categorization of diet responders and diet non-responders

In this intervention study the disease activity was assessed by means of a combined index called disease activity score from 28 joints (DAS28) [37]. The patients in the MD group who had a moderate or better clinical improvement from baseline to the end of the study, which is defined as a decrease of >0.6 in DAS28, were categorized as diet responders and the remaining MD patients as diet non-responders.

### Determination of the composition of fatty acids in serum phospholipids

#### Sampling

Blood samples for the analysis of fatty acids in serum phospholipids were taken at baseline and in weeks three and twelve. After a one-night fast, samples were collected in tubes without additives. The blood was cooled in ice water for 30 min before centrifugation at 1500 *g *for 15 min at +4°C. The serum was separated and stored at -70°C until analysis.

#### Folch extraction

Serum (1 ml) was extracted using methanol (2 ml) and chloroform (4 ml). Potassium chloride, 0,88%; (2 ml with the water content of the sample excluded) was added and the mixture was shaken thoroughly for 30 seconds before being allowed to settle. The aqueous layer was removed, one fourth of the volume of the organic phase of methanol-saline (1:1, v/v) was added and the washing procedure was repeated. The bottom layer containing the purified lipids was collected and the solvent was evaporated in a gentle stream of nitrogen at +30°C [38].

#### Separation of lipid classes using thin layer chromatography (TLC)

The purified lipids were solved in 40 μl chloroform containing 0,05% (v:v) of antioxidant butylated hydroxytoluene (BHT, 10% in ethanol (w:v)) and separated on silica gel plates 60, F_254 _(MERCK) by TLC, using petroleumether: diethyl ether: acetic acid (81:18:1). The different lipid classes were visualized using UV-light at 254 nm. The standards used were L-α-Phosphatidylcholine and L-α-Phosphatidylethanolamine, Larodan Fine Chemicals. The phospholipid spots were transferred into screw-capped glass tubes for preparation of fatty acid methyl esters [39].

#### Transesterification using sodium methoxide

The silica gel containing the lipids was dissolved in sodium-dried diethyl ether (0,5 ml) and methyl acetate (20 μl). Sodium methoxide, 0,5 M, in dry methanol (20 μl) was added. After 15 minutes at room temperature the reaction was stopped by the addition of acetic acid (2 μl). The solvent was evaporated in a gentle stream of nitrogen at +30°C. Iso-hexane with BHT (0,05%; 1 ml) was added and the mixture was centrifuged at 1500 *g *for 2 minutes. The supernatant layer was removed using a Pasteur pipette into a sample tube [40].

#### GC analysis

The composition of the fatty acids was determined using gas chromatography. Of the purified methylated sample 1 μl was injected into a HP 5890 series II gas chromatograph suited with a FID and an HP-FFAP capillary column (30 m × 0,25 mm × 0,25 μm). The He carrier gas flow was 13 ml/min. The detector temperature was 260°C. The injector split 1:50 at 220°C. A temperature programme was used with an initial temperature of 160°C held for 5 minutes, raised from 160 to 220°C at a rate of 2°C/min and 220°C held for 30 minutes.

The fatty acid methyl esters were identified by comparison with the retention times of the standards: GLC-68D and GLC-68A, Nu-Chek-Prep and Qualimix Fish 89 – 5540, Larodan Fine Chemicals.

The individual fatty acids were expressed as the percentage of total fatty acids (relative amount). The relative amounts were quantified by integrating the area under the peak and dividing the results by the total area of all fatty acids.

### Statistical methods

The statistical analyses were performed using SPSS for Windows version 11.0.1. The differences in fat intake between groups were analyzed using the Student's t-test for independent samples, when the variables were normally distributed. For fatty acids with skewed distributions the Mann-Whitney U test was used. As regards the reported consumption of food items and the composition of fatty acids in serum phospholipids, the Mann-Whitney U-test was performed to test differences between groups at baseline. For these variables, within group differences from baseline to week 12 were evaluated by means of the Wilcoxon signed ranks test.

The Spearman's rank correlation was used to evaluate the association between the reported dietary intake of fatty acids (assessed by diet history interviews including supplements) and their relative content in s-phospholipids. When calculating the correlation, the mean value of the results from week six and twelve were used regarding fatty acids in s-phospholipids, since the diet history interviews were performed between study weeks seven and twelve.

Spearman's rank correlation was also used to evaluate the association between the reported consumption of fish (estimated by means of the questionnaire) and the long chain n-3 fatty acids in s-phospholipids. For these assessments the change from baseline to week 12 was used.

All results were considered statistically significant at a two-tailed p-value of <0.05.

## List of abbreviations

AA, arachidonic acid; BMI, body mass index; CD, control diet; DAS 28, disease activity score from 28 joints; DHA, docosahexaenoic acid; EPA, eicosapentaenoic acid, E%, percentage of the energy intake; FIL, food intake level; LA, linoleic acid; α-LNA, α-Linolenic acid; MD, Mediterranean diet; MUFA, monounsaturated fatty acids; OA, oleic acid; PUFA, polyunsaturated fatty acids; RA, rheumatoid arthritis; SFA, saturated fatty acids

## Competing interests

The author(s) declare that they have no competing interests.

## Authors' contributions

LH participated in the conception and design, acquisition of data, data analysis, interpretation of data and the writing of the manuscript. IN participated in the data analysis, the critical revision of the manuscript and the writing of parts of the paper. LS participated in the conception and design, acquisition of data, interpretation of data and the critical revision of the manuscript. GJ participated in the conception and design, data analysis, interpretation of data, and the critical revision of the manuscript. All authors have read and approved the final manuscript.
